# Functional germline variants as potential co-oncogenes

**DOI:** 10.1038/s41523-017-0051-5

**Published:** 2017-11-22

**Authors:** Divyansh Agarwal, Christoph Nowak, Nancy R. Zhang, Lajos Pusztai, Christos Hatzis

**Affiliations:** 1 Department of Genomics and Computational Biology, Perelman School of Medicine, University of Pennsylvania, Philadelphia, PA USA; 2 Department of Statistics, The Wharton School, University of Pennsylvania, Philadelphia, PA USA; 3 Department of Medical Sciences, Molecular Epidemiology, Uppsala University, Uppsala, Sweden; 4 Department of Neurobiology, Care Sciences and Society, Karolinska Institutet, Huddinge, Sweden; 5 Department of Medicine, Breast Medical Oncology, Yale School of Medicine, Yale University, New Haven, CT USA

## Abstract

Germline variants that affect the expression or function of proteins contribute to phenotypic variation in humans and likely determine individual characteristics and susceptibility to diseases including cancer. A number of high penetrance germline variants that increase cancer risk have been identified and studied, but germline functional polymorphisms are not typically considered in the context of cancer biology, where the focus is primarily on somatic mutations. Yet, there is evidence from familial cancers indicating that specific cancer subtypes tend to arise in carriers of high-risk germline variants (e.g., triple negative breast cancers in mutated BRCA carriers), which suggests that pre-existing germline variants may determine which complementary somatic driver mutations are needed to drive tumorigenesis. Recent genome sequencing studies of large breast cancer cohorts reported only a handful of highly recurrent driver mutations, suggesting that different oncogenic events drive individual cancers. Here, we propose that germline polymorphisms can function as oncogenic modifiers, or co-oncogenes, and these determine what complementary subsequent somatic events are required for full malignant transformation. Therefore, we propose that germline aberrations should be considered together with somatic mutations to determine what genes drive cancer and how they may be targeted.

## Introduction

The genome of two individuals differs from one another, and from the reference human genome, in 20,000–25,000 non-synonymous single nucleotide polymorphisms (SNPs) in coding regions that affect the functions of hundreds of genes. If noncoding regions are included, up to 3.5–4.3 million SNPs show inter-individual variability.^1,2^ Phenotypically normal individuals can also carry several small and large scale structural DNA alterations that affect up to 0.7% of their genome.^2^ Collectively, the germline functional variants that individuals carry determine their unique features and influence susceptibility to disease. Cells in self-renewing tissues accumulate somatic mutations over time as a result of natural growth, and can evolve to malignant lesions when acquiring an initiating or driver mutation. These cancer initiating somatic mutations arise in the context of individual germline DNA polymorphisms and thus may be different for different cancers. Most genetic variants detected in any given cancer are germline variants, with solid tumors harboring only a few dozen to a few hundred additional somatic mutations that are considered potentially important in cancer biology.^3,4^

The causal implication of many germline variants in Mendelian diseases has long been recognized (http://omim.org/).^5^ Numerous cancer predisposing germline variants have also been identified in tumor suppressor genes (*TP53*, *RB*), in genes involved in DNA repair (e.g., *BRCA1*, *PALB2*, *MLH1*, *MSH2/6*, *CHEK2*, and *ATM*), cell proliferation (e.g., *PTEN*, *STK11*, *RET*, and *FGFR*) and cell adhesion (e.g., *CDH1*, *APC*).^6–9^ However, only a small proportion of breast cancer risk can be attributed to high penetrance germline mutations, with each individual SNP contributing only marginally to the risk of cancer. This suggests that the impact of any given variant on disease risk depends on co-occurrence of functional variants in different genes possibly acting in the same pathway, and that additional acquired somatic mutations (or epigenetic events) are required for initiation of malignant transformation.^10^ This phenomenon can even be observed in individuals who carry high-penetrance germline mutations such as BRCA1/2 carriers,^9,11^ in which additional germline variants can substantially influence the cancer risk conferred by a given germline BRCA mutation.^10^ The increasing availability of well-powered prospective population studies such as the UK Biobank of >500,000 persons (www.ukbiobank.ac.uk/) is likely to identify additional low-risk variants for cancer.

An unexpected observation from large-scale cancer sequencing efforts was the relative rarity of high frequency, recurrent somatic mutations in common solid tumors.^12^ In total, 37% of breast cancers carry somatic mutations in *TP53* and 36% in *PIK3CA*, the two most-frequently mutated genes, while most cancers display a variable assortment of low-frequency mutations in unique combinations. Yet, the majority (80%) of basal-like cancers have a *TP53* mutation compared to 9% with a *PIK3CA* mutation, but only 12% of liminal A cancers have *TP53* mutations compared to 45% who have *PIK3CA* mutations, suggesting a strong association between driver mutations and cancer subtype. A possible explanation could be that the effect of different mutations converges at the biological pathway level. Distinct genes may be affected in different individuals but the net biological impact is the dysregulation of an oncogenic pathway or biological process through the combined action of several mutations.^13^ Furthermore, we suggest that functional germline polymorphisms could in effect function as pre-existing driver “hits”, which together with complementary somatic mutations acquired at a later time act to dysregulate key pathways and to trigger full malignant transformation.

This hypothesis is a conceptual extension of Alfred Knudson’s two-hit model of a single oncogenic event.^14^ Knudson postulated that one germline and one somatic hit are required to disable both alleles of a tumor suppressor gene, which then leads to cancer formation.^14,15^ This hypothesis could be extended beyond consideration of only two alleles in the same gene to the dysregulation of oncogenic pathways.^13^ Similarly, activation of only one copy of an proto-oncogene may be required for carcinogenesis through a gain-of-function mutation or through amplification of the locus, which can occur potentially in concert with cooperative effects in the germline DNA of the oncogene or of related genes.

**Fig. 1:**
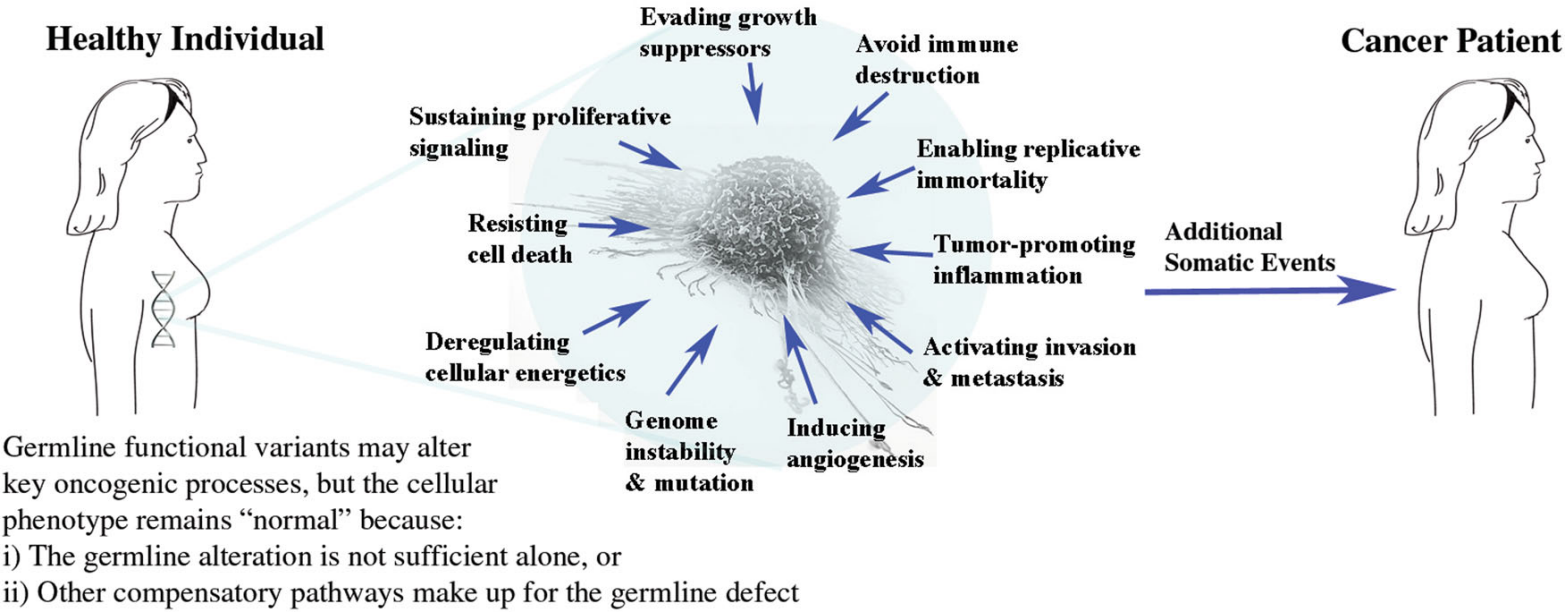
Germline variants may act as co-oncogenes in the dysregulation of biological processes that are the “hallmarks of cancer”

We therefore propose that key biological processes that are required for breast carcinogenesis^13^ can be altered through effects from the germline DNA, but that these events alone are not sufficient to initiate malignant transformation. Such germline abnormalities may remain silent because of compensatory pathways in normal tissues and lack of dysregulation in other complementary “hallmarks” of cancer until acquired somatic mutations disable these compensatory pathways or activate complementary oncogenic processes (Fig. 1). What constitutes a somatic cancer driver event in a particular patient is conditional on the collection of functional polymorphisms already present in her/his germline DNA. This hypothesis is consistent with the widely observed, but rarely discussed, cell-line-restricted transforming effects of many known oncogenes.^16,17^

## Experimental data supporting functional cooperation between germline SNPs and somatic mutations

There is a growing list of germline variants that have been shown experimentally to impact protein function without having a detectable association with disease risk, with several of these affecting genes implicated in cancer. For example, the M326I variant (rs3730089, variant allele frequency [VAF] of the A-allele 22%) in the p85α regulatory subunit of phosphatidylinositol 3-kinase (PI3K) results in constitutively increased activity of the PI3K pathway.^18^ Similarly, the L1016S variant (rs61733127, C-allele VAF 9%) in the PH domain and leucine-rich repeat protein phosphatase 2 (PHLPP2) leads to reduced phosphatase activity and increased signaling through the protein kinase B (AKT) and protein kinase C (PKC).^19^

The T1010I (rs56391007, T-allele VAF 1%) germline variant in MET (hepatocyte growth factor receptor) increases colony formation, cell migration, invasion, and in vivo tumor growth when introduced into MCF-10A immortalized breast epithelial cell line.^20^ Germline loss-of-function variants in PALB2 (partner and localizer of BRCA2) lead to predisposition to breast and pancreatic cancer. In mouse models, deletion of PALB2 led to mammary tumor formation with long latency. However, co-deletion of TP53 resulted in accelerated tumor formation, providing indirect support for synergistic interactions between a germline and a frequently observed somatic mutation.^21^

A recent analysis of germline and somatic DNA data from nearly 7000 patients from the cancer genome atlas (TCGA) identified almost 400 germline SNPs that were significantly associated with tumors of a particular type compared to all other cancer types.^22^ Fifteen of these markers had also been previously identified in GWAS studies, and listed in the National Human Genome Research Institute GWAS catalog, as being associated with significant cancer risk compared to nondiseased controls. This suggests that these germline loci are not only associated with significantly elevated cancer risk, but they also appear to influence the type and biology of the cancer that emerges. Furthermore, 17 germline loci were significantly associated with the somatic alteration rate of known cancer genes. These loci were not proximal to the affected cancer genes but converged to the same biological pathways,^22,23^ providing direct evidence for our hypothesis.

## Population-based indirect evidence

Past studies have shown that several sporadic cancers have a significant inherited component, and demonstrated genome-wide SNP relatedness in twelve cancer types.^24^ Based on the common variants of modest effect that influence breast cancer risk, polygenic risk scores using reported susceptibility loci from population-based studies were developed, and their associations with risks for BRCA1 and BRCA2 carriers were evaluated.^25–27^ These polygenic predictors suggest that polygenic risk scores based on common variants could provide clinically useful risk stratification and management of BRCA mutation carriers, and may provide a starting basis for biological interpretation of the confluence of genetic events that drive cancer.

Prognostic and predictive marker research has focused on somatic alterations found in cancer cells and on alterations in the tumor microenvironment. Recent large population studies, and particularly those based on Mendelian randomization (MR) designs to infer causal relationships between correlated variables, have revealed intriguing insights. The MR approach^28^ uses common genetic variants associated with an exposure as instruments to test for causal effects on an outcome. Since parental alleles are essentially allocated at random during meiosis, this “quasi-randomization” before birth minimizes bias from subsequent environmental factors and reverse causation, and is finding increasing application in polygenic traits.^29^ The role of germline variants, whose individually small effects on breast cancer are far from genome-wide significant, is demonstrated, for instance, in evaluation of adult height as a breast cancer risk factor: Zhang and colleagues^30^ found a causal effect of genetically predicted increase in height on breast cancer risk both in premenopausal and postmenopausal women, but restricted only to hormone-receptor positive breast cancer. Additionally, recent results from large-scale population studies suggest that prognosis of early-stage breast cancer tends to cluster in families; the survival of first-degree relatives is statistically more similar than expected by chance after adjusting for tumor and treatment characteristics.^31,32^ These observations suggest that cancer occurrence and survival are influenced not only by somatic mutations but also by hereditary components whose contribution may be missed in traditional genome-wide association studies.

For the vast majority of SNPs discovered in epidemiologic studies, there is no functional evidence to demonstrate their impact on biological processes and pathways. In principle, by “functional” we refer to any germline variant that is predicted to affect protein function, regardless of the magnitude of altered protein activity, or whether the effect manifests in a clear phenotype or disease. In the illustrative analysis that follows, we focused on genetic variants predicted to have large (i.e., deleterious) effects on protein function. Since most trait-associated SNPs are in noncoding regions of the genome, efforts to identify causal genes through traditional methods such as targeted resequencing and single-gene knock-outs have been challenging. Yet, recent high-throughput, high-specificity gene-editing approaches enabled by CRISPR/Cas9 are expected to dramatically increase the catalog of germline variants with biologically validated function.^33^

We performed an exploratory analysis to examine whether predicted high functional impact germline SNPs co-occur with somatic mutations in different breast cancer subtypes. We filtered the germline SNPs reported in the TCGA breast cancer cohort to retain 8598 germline variants with CADD *C*-score ≥20, corresponding to the 1% most deleterious variants in the human genome (details on data processing steps are given in Table 1 notes). To limit the number of comparisons, we tested for co-occurrence of these high functional impact germline variants with somatic mutations occurring only in canonical cancer-causing genes in the catalog of somatic mutations in cancer (COSMIC; http://cancer.sanger.ac.uk)^34^ database using a two-sided Fisher exact test. This analysis identified several germline SNP—somatic mutation pairs that co-occurred more frequently than expected by chance, mostly within specific breast cancer subtypes (Table 1). These observations are consistent with the hypothesis of synergistic interactions between functional germline SNPs and rare somatic mutations in oncogenesis.

**Table 1:** Significant associations between germline variants and somatic mutations detected in the TCGA breast cancer data at a false discovery rate ≤12%

Somatic gene	Germline variant					Two-sided Fisher exact test *p*-value	Direction
	rsID	Gene	SNP type	VAF 1000 genomes	VAF TCGA		
Luminal A (*N* = 210)							
SSPO	rs45551636	PALB2	Missense	0.008	0.028	4 × 10^–6^	Co-mutation
MACF1	rs3923647	TLR1	Missense	0.029	0.025	4 × 10^–6^	Co-mutation
Luminal B (*N* = 115)							
USH2A	rs8140287	ISX	Missense	0.031	0.045	9 × 10^–5^	Co-mutation
ATP10B	rs2273137	NOP56	Missense	0.118	0.084	8 × 10^–5^	Co-mutation
PIK3CA	rs12099177	MMP27	Missense	0.168	0.081	8 × 10^–5^	Co-exclusion
SYNE1	rs34605667	MPDZ	Missense	0.014	0.037	5 × 10^–5^	Co-mutation
Basal (*N* = 54)							
USH2A	rs17848337	SREBF2	Synonymous	0.051	0.051	1 × 10^–4^	Co-mutation
TP53	rs55695858	OBP2A	Missense	0.173	0.224	1 × 10^–4^	Co-mutation
HER2-enriched (*N* = 90)							
EYS	rs2066518	SMARCAL1	Missense	0.073	0.026	2 × 10^–4^	Co-mutation
PIK3CA	rs3774372	ULK4	Missense	0.173	0.176	1 × 10^–4^	Co-mutation

The germline variants included in this analysis were the 8598 CADD* predicted-deleterious SNPs (*C*-score ≥ 20). Only somatic mutations in canonical cancer genes listed in COSMIC and OMIM that had mutations in ≥2% of breast cancer cases were included (1649 mutations in 71 genes). Somatic mutations were aggregated at gene level for statistical comparison with individual SNPs. Molecular subtype annotation was available through the TCGA

(*) Combined annotation-dependent depletion^36^

There are several caveats in this exploratory analysis, including technical issues related to the germline variant calls in the TCGA^35^ and the heterogeneity in sequencing depth and quality control filtering between germline and somatic mutation calls. These analyzes will need to be repeated in larger cohorts.

## Conclusions

Most genomic analyzes of sporadic cancers discard the vast number of germline variants that are detected in a cancer as functionally irrelevant. Ignoring the biological contribution of these variants may lead to an overly simplistic view of the cancer genome. Recent data provide evidence suggesting that neoplastic phenotypes can emerge through cooperation between functionally important germline loci and somatic alterations. We propose that this is a wide-spread phenomenon where tolerated germline SNPs can act as co-oncogenes or co-tumor suppressors and functionally synergize with somatic mutations to induce the neoplastic phenotype. Biological pathways can be altered at multiple different levels and alterations in different member genes could cause similar phenotypic effects. Therefore, the possible combination of synergistic germline and somatic mutations is vast. This could explain the relative lack of high frequency recurrent somatic events in most solid tumors and would also suggest that, when studied individually, co-oncogenic SNPs may not be identified as cancer predisposing variants. This hypothesis is testable in the laboratory; for instance, Table 1 reports germline SNP-somatic mutation pairs whose functional synergy could be evaluated experimentally. More nuanced understanding of interactions between germline and somatic genetic events could fundamentally change our conceptions about breast cancer biology and breast cancer prevention.
